# An Optimal Control Method for Maximizing the Efficiency of Direct Drive Ocean Wave Energy Extraction System

**DOI:** 10.1155/2014/480916

**Published:** 2014-07-24

**Authors:** Zhongxian Chen, Haitao Yu, Cheng Wen

**Affiliations:** Engineering Research Center of Motion Control of Ministry of Education, Southeast University, Nanjing 210096, China

## Abstract

The goal of direct drive ocean wave energy extraction system is to convert ocean wave energy into electricity. The problem explored in this paper is the design and optimal control for the direct drive ocean wave energy extraction system. An optimal control method based on internal model proportion integration differentiation (IM-PID) is proposed in this paper though most of ocean wave energy extraction systems are optimized by the structure, weight, and material. With this control method, the heavy speed of outer heavy buoy of the energy extraction system is in resonance with incident wave, and the system efficiency is largely improved. Validity of the proposed optimal control method is verified in both regular and irregular ocean waves, and it is shown that IM-PID control method is optimal in that it maximizes the energy conversion efficiency. In addition, the anti-interference ability of IM-PID control method has been assessed, and the results show that the IM-PID control method has good robustness, high precision, and strong anti-interference ability.

## 1. Introduction

Ocean wave energy is higher in energy density than other forms of renewable resource such as wind, solar energy, and tides [1]. In the past thirty years, a diversity of ocean wave energy extraction systems have been investigated [2–6]. Generally, ocean wave energy extraction systems can be classified into single-body floating buoy, two-body floating buoys, and multibody floating buoys by the number of floating buoys [7]. This paper focuses on two-body heavy buoys and PMTLG system with which ocean wave energy is converted into electricity directly.

In order to maximize the efficiency of the ocean wave energy extraction system, some optimal control methods are proposed. The method of complex-conjugate control is proposed to optimize the dimensions of heavy buoy and to maximize energy extraction from regular waves (sinusoidal waves) [8, 9]. However, this optimal control method can not be applied in the irregular waves (ocean waves) because the dimensions of heavy buoys are noncausal. To accomplish the optimal operation in ocean waves, a reactive control method based on the winding current of generators has been proposed [10]. Ideally, it is possible to adjust the winding current of generators to achieve the resonance between heavy buoy and ocean incident waves. However, the overload of winding current due to ocean waves' high speed, especially with hurricane or typhoon, has not been taken into consideration in the reactive control method. In addition, the efficiency of generators will be decreased by adjusting the winding current.

Simple control and high efficiency make IM-PID an attractive candidate for the optimal control of the ocean wave energy extraction system. In comparison with the conventional PID (proportion integration differentiation) control method, the IM-PID control method can be implemented by adjusting the filter coefficient *ε*, while the conventional PID control method needs to adjust the three parameters: proportion *P*, integration *I*, and differentiation *D*. In addition, software and hardware circuit development make the adoption of IM-PID control method possible.

Therefore, this paper proposes the IM-PID control method to optimize the dynamic performance of direct drive ocean wave energy extraction system. The IM-PID control method involves the anti-interference ability assessment, while lots of pervious researches focus on the optimization of generator or heavy buoys. The paper is organized as follows.  Section 1 introduces the subject and a review of optimal control methods for the ocean wave energy extraction system. In Section 2, hydrodynamic analysis for heavy buoys, including the electromagnetic force of PMTLG, is presented.  Section 3 is for the proposed IM-PID control method to maximize the conversion of ocean wave energy into electricity. Results are in Section 4 and Section 5 is the conclusion.

## 2. Hydrodynamic and Electromagnetic Analysis

**Figure 1:**
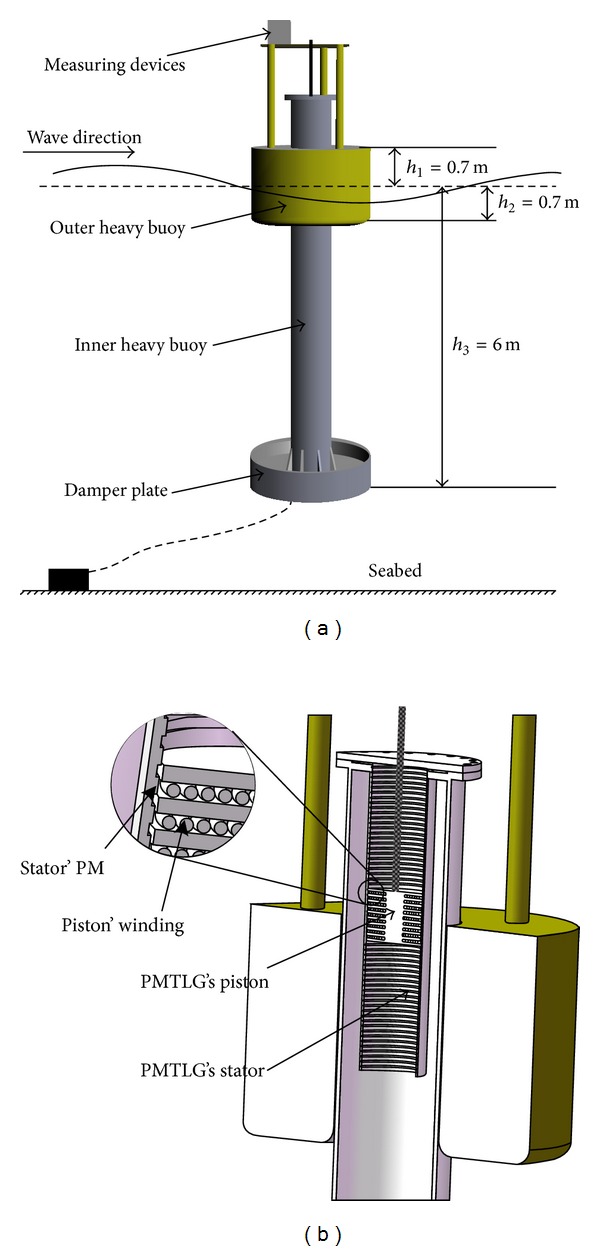
Direct drive ocean wave energy extraction system: (a) general scheme; (b) installation location of the PMTLG.

Direct drive ocean wave energy extraction system converts ocean wave energy into electricity directly without any intermediate links, such as pneumatics, hydraulic, linear-to-rotary conversion, or others.  Figure 1 depicts a general scheme of the direct drive ocean wave energy extraction system where the oscillating system is made up of two heavy buoys, a PMTLG, a damper plate, and some other devices.

### 2.1. Hydrodynamic Analysis of Heavy Buoy

As a mathematical description of the heavy buoys, they are assumed to oscillate only in the vertical direction. Assumptions are further made that the dimensions of heavy buoys are smaller than incident wavelength, and the fluid is incompressible. Then, the method of frequency domain analysis and linear theory are adopted to describe the reciprocating oscillation of heavy buoys. The motion equation of the heavy buoys can be described as [11]

(1) $$\begin{array}{c} m{\hat{a}}_{z}={\hat{F}}_{z}+{\hat{F}}_{r}+{\hat{F}}_{b}+{\hat{F}}_{f}, \end{array}$$

where *m* is the heavy buoy's weight and ${\hat{a}}_{z}$ is the heavy buoy's acceleration. ${\hat{F}}_{z}$ is the vertical wave force, which is the result of incident ocean waves, and can be written as

(2) $$\begin{array}{c} {\hat{F}}_{z}=\left[\rho gS_{w}-\omega^{2}\rho V\left(1+\mu_{z}\right)\right]\hat{\eta }, \end{array}$$

where *ρ* is the density of ocean water, *g* is the acceleration of gravity, *S*_*w*_ is the water plane area of heavy buoy, *ω* is the angular frequency, *V* is the volume of displaced ocean water of heavy buoy, *μ*_*z*_ is the added mass coefficient, and $\hat{\eta }$ is the complex amplitude of incident ocean waves [12, 13].

Further, ${\hat{F}}_{r}$ is the radiation force resulting from the reciprocating oscillation of heavy buoy, ${\hat{F}}_{b}$ is the hydrostatic buoyancy force, and ${\hat{F}}_{f}$ is the friction force. ${\hat{F}}_{r},{\hat{F}}_{b},{\hat{F}}_{f}$ can be described as

(3) $$\begin{array}{c} {\hat{F}}_{r}=\left(\omega^{2}m_{z}-i\omega R_{z}\right)\frac{{\hat{a}}_{z}}{-\omega^{2}},\\ {\hat{F}}_{b}=-\rho gS_{w}\frac{{\hat{a}}_{z}}{-\omega^{2}},\\ {\hat{F}}_{f}=-i\omega R_{f}\frac{{\hat{a}}_{z}}{-\omega^{2}}, \end{array}$$

where *m*_*z*_ is the heavy buoy's added mass, *R*_*z*_ is the heavy buoy's radiation damping coefficient [12, 13], and *R*_*f*_ is the friction resistance coefficient of direct drive ocean wave energy extraction system.

Substituting (2) and (3) into (1), and according to the complex representation ${\hat{a}}_{z}=i\omega {\hat{v}}_{z}=-\omega^{2}{\hat{s}}_{z}$, ${\hat{v}}_{z}$ and ${\hat{s}}_{z}$ are the heavy speed (the speed in the vertical direction) and the displacement of heavy buoy, respectively. Therefore, the heavy speed and displacement of heavy buoy can be written as

(4) $$\begin{array}{c} {\hat{v}}_{z}=\frac{\left[\rho gS_{w}-\omega^{2}\rho V\left(1+\mu_{z}\right)\right]\hat{\eta }}{i\omega \left[m+m_{z}\right]+\left[R_{f}+R_{z}\right]+\left(\rho gS_{w}/i\omega \right)}, \end{array}$$

(5) $$\begin{array}{c} {\hat{s}}_{z}=\frac{\left[\rho gS_{w}-\omega^{2}\rho V\left(1+\mu_{z}\right)\right]\hat{\eta }}{-\omega^{2}\left[m+m_{z}\right]+i\omega \left[R_{f}+R_{z}\right]+\rho gS_{w}}. \end{array}$$

For the certain angular frequency *ω*, (4) indicates that the heavy speed of outer heavy buoy can be in resonance with incident wave by modifying its weight, which means the imaginary part of (4) is zero.

**Figure 2:**
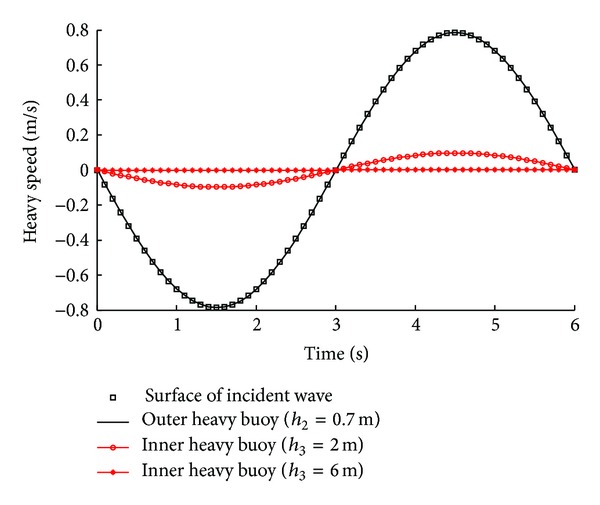
The heavy speed of heavy buoys (without consideration of the PMTLG's force). The diameter of outer heavy buoy is 2.4 m, the diameter of inner heavy buoy is 0.86 m, and the depths in water *h*_2_ and *h*_3_ are shown in Figure 1(a).

It is assumed that the ocean waves propagate regularly in sinusoidal trace; the wave period is 6 seconds and the wave height is 1.5 meters. From the above hydrodynamic analysis of heavy buoy, the heavy speeds of outer and inner heavy buoys are shown in Figure 2. From Figure 2, it can be seen that the heavy speed of outer heavy buoy is in approximate resonance with incident wave (with the adoption of weight optimization of outer heavy buoy). It also verified that the heavy speed of inner heavy buoy depends on its depth in water *h*_3_, and the inner heavy buoy is considered to be stationary when *h*_3_ = 6 m. The relative heavy speed between outer and inner heavy buoys drives the PMTLG to generate electricity.

In practice, the method of weight optimization of outer heavy buoy can not be applied in direct drive ocean wave energy extraction system due to the various angular frequencies *ω* of ocean waves and the inconstant force of PMTLG (see Section 2.2).

### 2.2. Electromagnetic Analysis of PMTLG

PMTLG is one of the most important devices of ocean wave energy extraction system. In this paper, a three-phase PMTLG is proposed and installed in the inner heavy buoy (see Figure 1(b)).

At no-load condition, the only force derived from PMTLG is detent force [14, 15]. Detent force, which resulted from the magnetic attraction between PMTLG's piston and permanent magnets (fixed on PMTLG's stator), plays a significant role in the dynamic performance of direct drive ocean wave energy extraction system. According to the Maxwell stress tensor method [16], the governing equation of detent force can be expressed as

(6) $$\begin{array}{c} F_{u}=\overset{}{\underset{A}{\oint }}\frac{1}{2\mu_{0}}\left(B_{n}-B_{t}\right)dA\cdot t+\frac{1}{\mu_{0}}B_{n}B_{t}dA\cdot n, \end{array}$$

where *A* is the integral area of permanent magnets in line with PMTLG's piston, *μ*_0_ is the air permeability, *B* is the flux density, and *n* and *t* are the normal direction unit vectors and tangential direction unit vectors on the integral area, respectively.

At load condition, a load force is added to PMTLG besides the detent force. Actually, the amplitude and frequency of load force depend on the winding current ${\hat{i}}_{q}$, which is one of the current components in the *dq*0 reference frame. The *dq*0 components of PMTLG can be written as follows [17, 18]:

(7) $$\begin{array}{c} {\hat{v}}_{d}=R_{S}{\hat{i}}_{d}+L_{S}\frac{d{\hat{i}}_{d}}{dt}-\omega_{G}L_{S}{\hat{i}}_{q},\\ {\hat{v}}_{q}=R_{S}{\hat{i}}_{q}+L_{S}\frac{d{\hat{i}}_{q}}{dt}+\omega_{G}L_{S}{\hat{i}}_{d}+\omega_{G}\psi ,\\ {\hat{v}}_{0}=R_{S}{\hat{i}}_{0}+L_{S}\frac{d{\hat{i}}_{0}}{dt}, \end{array}$$

where *R*_*S*_ is the winding resistance, *L*_*S*_ is the winding inductance, *ω*_*G*_ is the PMTLG's angular frequency, and *ψ* is the flux linkage caused by stator's permanent magnets. Besides, ${\hat{v}}_{d}$, ${\hat{v}}_{q}$, ${\hat{i}}_{d}$, ${\hat{i}}_{q}$, ${\hat{v}}_{0}$, and ${\hat{i}}_{0}$ are the parameters based on *dq*0 reference frame. It is assumed that there is delta connection; then, the sum of three-phase voltages is zero and the zero sequence voltage ${\hat{v}}_{0}$ is null. Therefore, the ocean wave energy that PMTLG extracted may be expressed as [19]

(8) $$\begin{array}{c} {\hat{P}}_{G}=\frac{3}{2}\frac{\pi \psi }{\tau }{\hat{v}}_{z}{\hat{i}}_{q}. \end{array}$$

According to ${\hat{P}}_{G}=-{\hat{F}}_{G}{\hat{v}}_{z}$ and (8), the PMTLG's load force ${\hat{F}}_{G}$ can be obtained as

(9) $$\begin{array}{c} {\hat{F}}_{G}=-\frac{3}{2}\frac{\pi \psi }{\tau }{\hat{i}}_{q}, \end{array}$$

where *τ* is the pole pitch of PMTLG. Considering the detent force *F*_*u*_ and the load force ${\hat{F}}_{G}$, the heavy speed and displacement of heavy buoy are rewritten as

(10) $$\begin{array}{c} {\hat{v}}_{z}=\frac{\left[\rho gS_{w}-\omega^{2}\rho V\left(1+\mu_{z}\right)\right]\hat{\eta }+{\hat{F}}_{u}+{\hat{F}}_{G}}{i\omega \left[m+m_{z}\right]+\left[R_{f}+R_{z}\right]+\left(S_{w}/i\omega \right)}, \end{array}$$

(11) $$\begin{array}{c} {\hat{s}}_{z}=\frac{\left[\rho gS_{w}-\omega^{2}\rho V\left(1+\mu_{z}\right)\right]\hat{\eta }+{\hat{F}}_{u}+{\hat{F}}_{G}}{-\omega^{2}\left[m+m_{z}\right]+i\omega \left[R_{f}+R_{z}\right]+\rho gS_{w}}, \end{array}$$

where ${\hat{F}}_{u}$ is the Fourier representation of detent force *F*_*u*_.

The detent force ${\hat{F}}_{u}$ only depends on the relative displacement between PMTLG's piston and permanent magnets, and load force ${\hat{F}}_{G}$ only relies on the parameters *τ*, *ψ*, and ${\hat{i}}_{q}$; thus, the method of weight optimization of heavy buoy cannot be employed in direct drive ocean wave energy extraction system. Besides, the linear equations (10) and (11) are only proper for constant frequency. This is because the added mass *m*_*z*_ and radiation damping coefficient *R*_*z*_ are decided by the angular frequency *ω* [12, 13], and the ocean waves' angular frequency *ω* is inconstant.

## 3. IM-PID Controller

### 3.1. IM-PID Control Model

**Figure 3:**
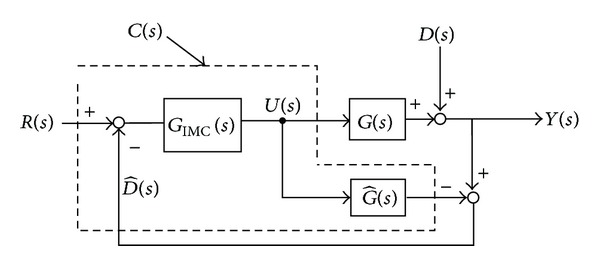
Block diagram of IM-PID.

IM-PID is practical in optimizing the dynamic performance of direct drive ocean wave energy extraction system, which has advantages of good robustness, high precision, and anti-interference ability.  Figure 3 depicts the block diagram of IM-PID, and the variables are described as follows:

 
* G*(*s*) is the plant (outer heavy buoy) to control;
 
* *
$\hat{G}\left(s\right)$ is the mathematical model of *G*(*s*);
 
* G*_IMC_(*s*) is IM-PID controller;
 
* C*(*s*) is feedback controller;
 
* Y*(*s*) is process output;
 
* R*(*s*) is reference input (the heavy speed of ocean surface waves);
 
* D*(*s*) is an unknown disturbance affecting the system.

In Figure 3, the consequent control signal *U*(*s*) can be written as

(12) U(s)=[R(s)−D^(s)]GIMC(s)=[R(s)−(G(s)−G^(s))U(s)−D(s)]GIMC(s).

Thus,

(13) $$\begin{array}{c} U\left(s\right)=\frac{\left[R\left(s\right)-D\left(s\right)\right]G_{\text{IMC}}\left(s\right)}{1+\left(G\left(s\right)-\hat{G}\left(s\right)\right)G_{\text{IMC}}\left(s\right)}. \end{array}$$

Since *Y*(*s*) = *G*(*s*)*U*(*s*) + *D*(*s*), the closed loop transfer function of IM-PID controller is

(14) Y(s)=G(s)GIMC(s)1+(G(s)−G^(s))GIMC(s)R(s) +G(s)[1−GIMC(s)G^(s)]1+(G(s)−G^(s))GIMC(s)D(s).

From Figure 3, the function of feedback controller is obtained as follows:

(15) $$\begin{array}{c} C\left(s\right)=\frac{G_{\text{IMC}}\left(s\right)}{1-G_{\text{IMC}}\left(s\right)\hat{G}\left(s\right)}. \end{array}$$

### 3.2. IM-PID Controller Design

Actually, the heavy speed of ocean waves can be described by a second-order linear approximate model [20]. Therefore, the controlled plant *G*(*s*) (outer heavy buoy) may be written as

(16) $$\begin{array}{c} G\left(s\right)=\frac{K_{P}}{\left(T_{1}s+1\right)\left(T_{2}s+1\right)}, \end{array}$$

where *T*_1_, *T*_2_, and *K*_*p*_ are the parameters of controlled plant. From (14), it can be concluded that when $G\left(s\right)=\hat{G}\left(s\right)$ and $G_{\text{IMC}}\left(s\right)={\hat{G}}^{-1}\left(s\right)$, then *Y*(*s*) = *R*(*s*). Therefore, it is assumed that $G\left(s\right)=\hat{G}\left(s\right)$; then, the dynamic performance of direct drive ocean wave energy extraction system can be controlled by modifying IM-PID controller *G*_IMC_(*s*).

Given the mathematical model of control plant $\hat{G}\left(s\right)$, the design of IM-PID controller *G*_IMC_(*s*) can be described as follows.

(1) Decompose the mathematical model of control plant $\hat{G}\left(s\right)$ into “invertible” and “noninvertible” components as follows:
  (17) $$\begin{array}{c} \hat{G}\left(s\right)={\hat{G}}_{+}\left(s\right){\hat{G}}_{-}\left(s\right); \end{array}$$
  the “invertible” component ${\hat{G}}_{+}\left(s\right)$ is an all-pass filter and ${\hat{G}}_{+}\left(s\right)=1$, for all *s*. Then, the “noninvertible” component ${\hat{G}}_{-}\left(s\right)$ can be obtained as
  (18) $$\begin{array}{c} {\hat{G}}_{-}\left(s\right)=\hat{G}\left(s\right)=\frac{K_{P}}{\left(T_{1}s+1\right)\left(T_{2}s+1\right)}. \end{array}$$
(2) Describe the IM-PID controller *G*_IMC_(*s*) as
  (19) $$\begin{array}{c} G_{\text{IMC}}\left(s\right)={\hat{G}}_{-}^{-1}\left(s\right)Q\left(s\right), \end{array}$$
 where *Q*(*s*) = 1/(*εs*+1)^2^ is a second-order filter (*ε* is the filter coefficient). Then, the IM-PID controller *G*_IMC_(*s*) and feedback controller function *C*(*s*) turn into
  (20) $$\begin{array}{c} G_{\text{IMC}}\left(s\right)=\frac{\left(T_{1}s+1\right)\left(T_{2}s+1\right)}{K_{P}{\left(\varepsilon s+1\right)}^{2}}, \end{array}$$
  (21) $$\begin{array}{c} C\left(s\right)=\frac{G_{\text{IMC}}\left(s\right)}{1-G_{\text{IMC}}\left(s\right)\hat{G}\left(s\right)}=K\left(1+\frac{1}{T_{i}s}+T_{d}s\right)\frac{1}{T_{f}s+1}, \end{array}$$
 where *K* = (*T*_1_ + *T*_2_)/2*K*_*P*_ *ε*, *T*_*i*_ = *T*_1_ + *T*_2_, *T*_*d*_ = *T*_1_ *T*_2_/(*T*_1_ + *T*_2_), and *T*_*f*_ = *ε*/2.

Parameters *T*_1_, *T*_2_, and *K*_*p*_ are known by the controlled plant *G*(*s*); thus, the dynamic performance of direct drive ocean wave energy extraction system can be optimized by designing *G*_IMC_(*s*) and *C*(*s*) through changing the filter coefficient *ε*.

## 4. Results

**Figure 4:**
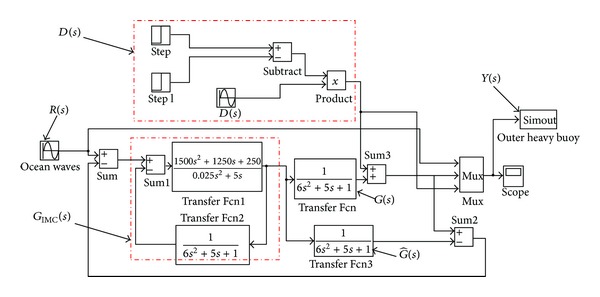
IM-PID control model for the direct drive ocean wave energy extraction system.

For the general outer heavy buoy geometry described in Figure 1(a), a second-order linear approximate plant model is written as *G*(*s*) = 1/[(2*s* + 1)(3*s* + 1)].  Figure 4 shows an overview of IM-PID control model for the direct drive ocean wave energy extraction system (the filter coefficient *ε* = 0.01). In Figure 4, the heavy speed of ocean surface wave *R*(*s*) is generated by applying the sinusoidal wave or Fourier representation to function signal generator and importing it into IM-PID controller *G*_IMC_(*s*). The design of IM-PID controller *G*_IMC_(*s*) is based on the controlled plant *G*(*s*) and filter coefficient *ε*, and the disturbance signal *D*(*s*) is generated by functional calculation.

**Figure 5:**
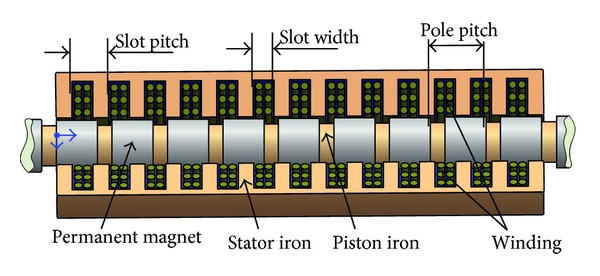
The basic structure of PMTLG.

**Figure 6:**
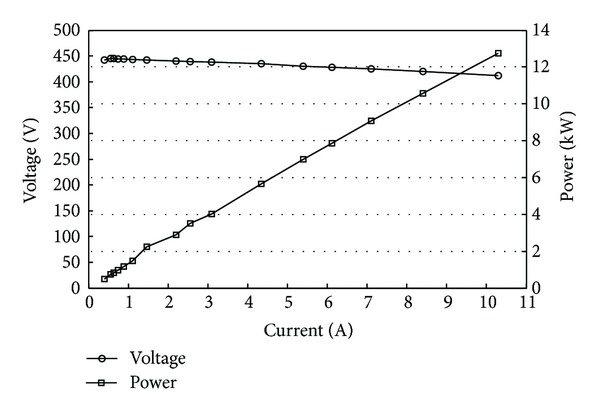
The load characteristics of PMTLG (without consideration of the coreless).

The basic structure of PMTLG proposed in this paper is presented in Figure 5, and its main parameters are shown in Table 1.  Figure 6 shows the load characteristics of PMTLG (the piston's velocity is 1 m/s). From Figure 6, it can be seen that the output power of PMTLG is proportional to the current because the voltage changes a little while the load resistance varies. In the case of rate load, the PMTLG's current is 6.12 A, the resistance is 70 Ω, the output power is 7.86 kW, and the copper loss is 561 W. The load characteristics of PMTLG decide the maximum wave height that PMTLG can be resisted (see Figure 8).

### 4.1. In Regular Ocean Waves

**Figure 7:**
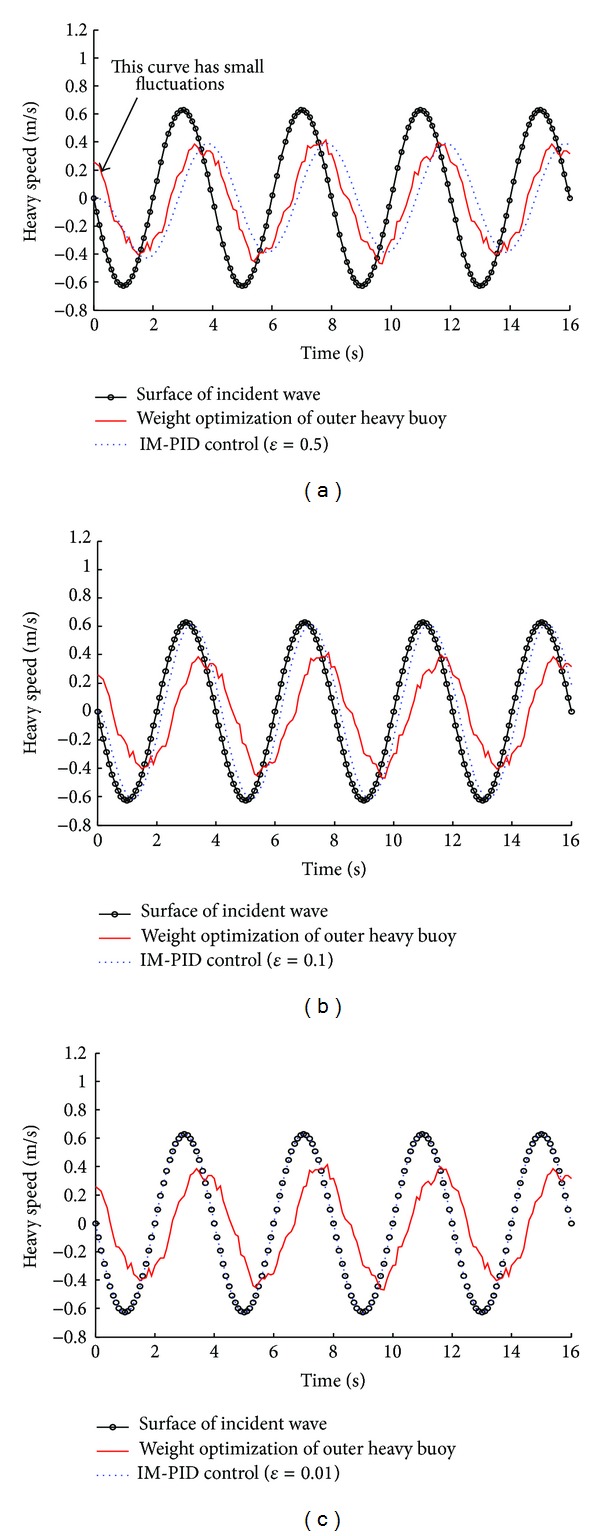
The heavy speed of outer heavy buoy in regular waves (at load condition): the wave period is 4 s and the wave height is 0.399 m.

For the regular ocean waves, it is assumed that the wave period is 4 s and the wave height is 0.399 m. At load condition, the heavy speed of outer heavy buoy is shown in Figure 7 when the IM-PID control method and the outer heavy buoy's weight optimization method are applied. If the filter coefficient *ε* = 0.5, the outer heavy buoy's amplitude and phase are less than or lag behind the surface of incident wave, which is similar to the outer heavy buoy's weight optimization method. When the filter coefficient *ε* = 0.01, the outer heavy buoy's amplitude and phase are in agreement with the surface of incident wave. Therefore, the smaller *ε* is, the more wave energy can be obtained, because the outer heavy buoy is in resonance with the incident ocean waves. In Figure 7, the small fluctuations of heavy speed by the outer heavy buoy's weight optimization method are due to the PMTLG's detent force and load force.

**Figure 8:**
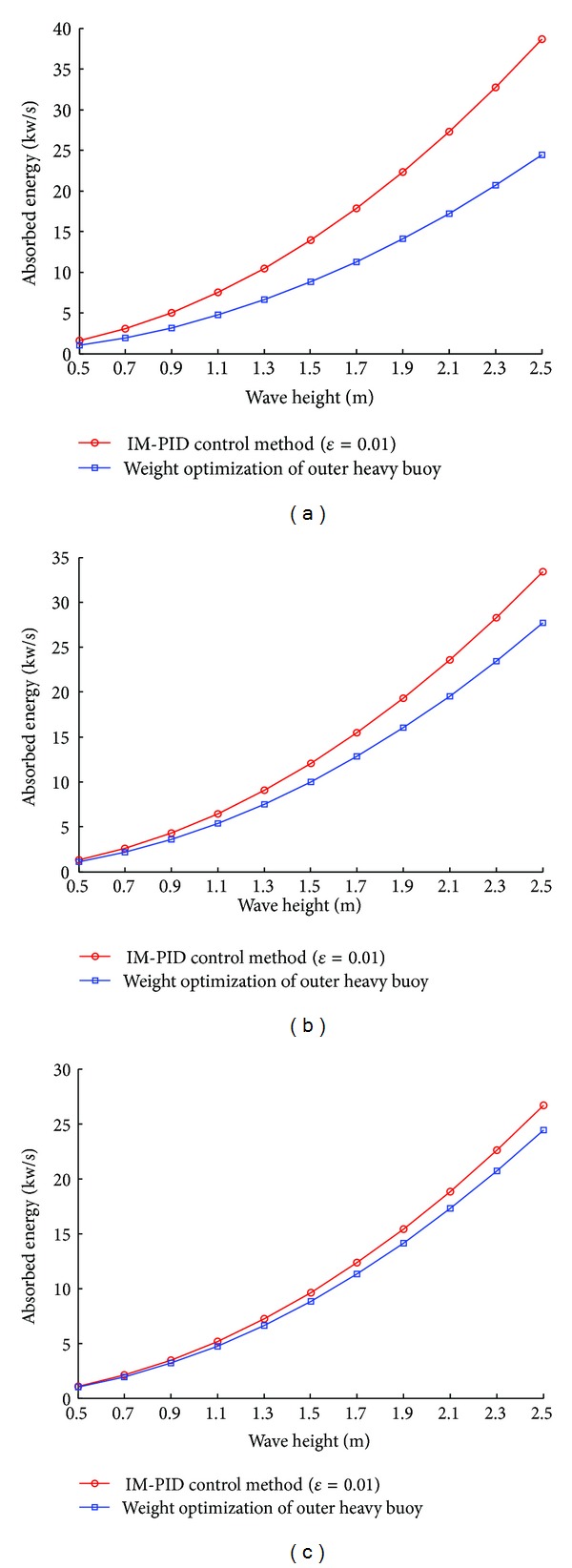
The average ocean wave energy absorption by each method: (a) the wave period is 4 s; (b) the wave period is 6 s; (c) the wave period is 8 s.

Figure 8 shows the average ocean wave energy that can be absorbed by the outer heavy buoy with the wave height and the wave period considered. Compared with the method of weight optimization of outer heavy buoy, the IM-PID control method has better performance in the ocean wave energy absorption.

### 4.2. In Irregular Ocean Waves

In most cases, the amplitude and frequency of ocean waves are irregular, and the added mass and damping coefficient of outer heavy buoy are not constant (caused by the irregular ocean waves' frequencies). Therefore, the method of weight optimization of heavy buoy can not be applied in the direct drive ocean wave energy extraction system.

**Figure 9:**
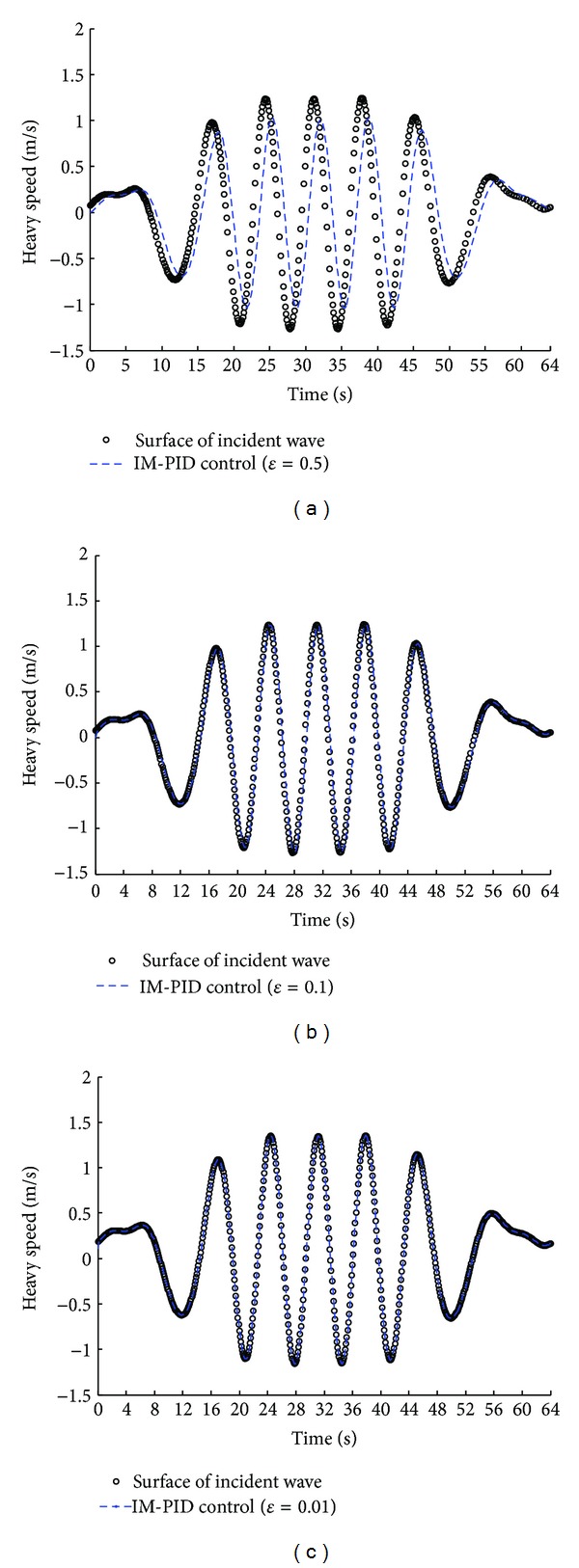
The heavy speed of outer heavy buoy in irregular waves (at load condition): (a)  *ε* = 0.5; (b)  *ε* = 0.1; (c)  *ε* = 0.01.

Fortunately, the method of IM-PID optimal control can be applied in the direct drive ocean wave energy extraction system.  Figure 9 depicts the optimization results of IM-PID control method based on a heavy speed time series of ocean surface waves (in the Yellow Sea at Lianyungang, China). Results on the real ocean surface waves' heavy speed data illustrate how an accurate control of the direct drive ocean wave energy extraction system can be realized by a simple IM-PID optimal control method based on the filter coefficient *ε*.

### 4.3. Anti-Interference Ability of the IM-PID Control Method

**Figure 10:**
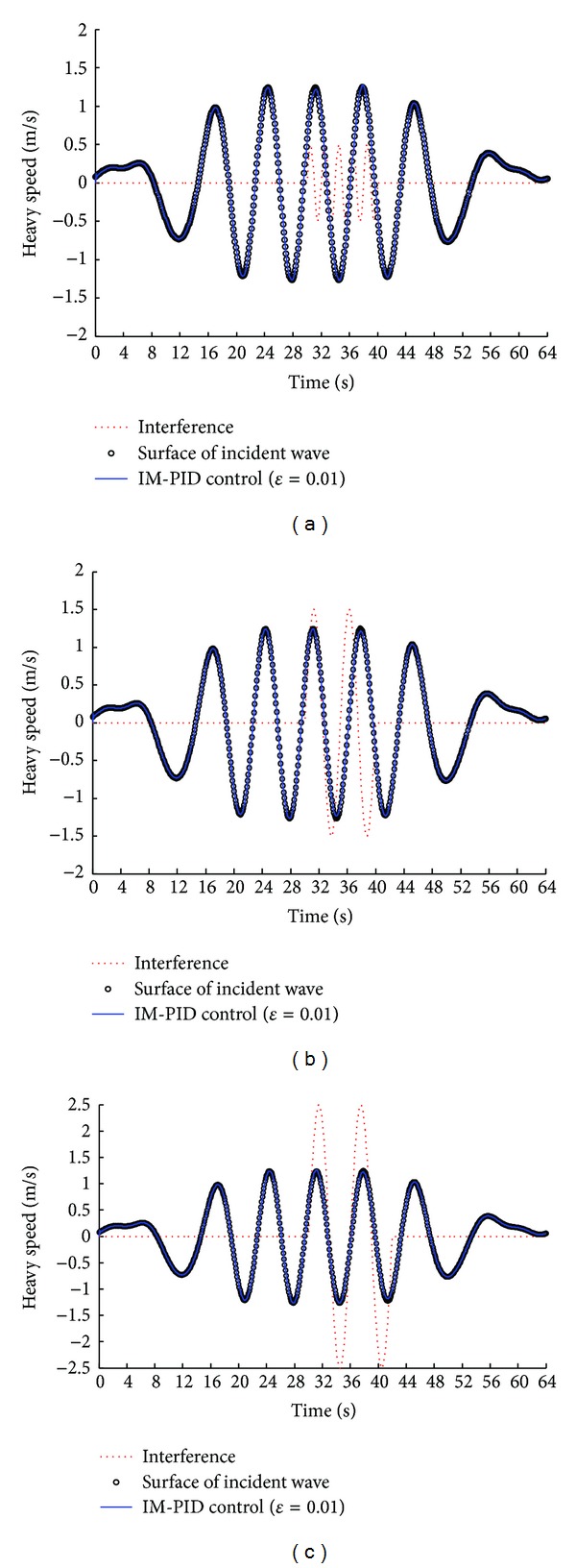
Anti-interference ability of the IM-PID control method in the irregular ocean waves: (a) the interference period is 2 s and the amplitude is 0.5 m/s; (b) the interference period is 5 s and the amplitude is 1.5 m/s; (c) the interference period is 6 s and the amplitude is 2.5 m/s.

Occasionally, the direct drive ocean wave energy extraction system will suffer from some interference such as breaking ocean waves and winds.  Figure 10 shows the anti-interference ability of IM-PID control method at load condition (the filter coefficient *ε* = 0.01). The results prove that the IM-PID control method has good robustness, high precision, and strong anti-interference ability. In addition, Figure 10 indicates that the IM-PID control method can resist different period (0~6 s) and amplitude (0~2.5 m/s) of interference.

## 5. Conclusions

In this paper, the anti-interference ability has been taken into account in the IM-PID optimal control method for direct drive ocean wave energy extraction system. It has been shown that the IM-PID optimal control method can be accomplished by adjusting the only filter coefficient *ε*, while the conventional PID control method needs to adjust the three parameters: proportion *P*, integration *I*, and differentiation *D*.

The proposed IM-PID optimal control method maximizes the energy conversion from the ocean waves to electricity and improves the dynamic performance of direct drive ocean wave energy extraction system. In addition, the IM-PID optimal control method has good robustness and high precision, so the stability of direct drive ocean wave energy extraction system increases.

Additionally, PMTLG optimization including detent force reduction has been achieved in other papers, which benefits the proposed IM-PID optimal control method implementation.

## Figures and Tables

**Table 1:** Main parameters of PMTLG.

	Item	Value (unit)
Stator	Pole pitch	49 (mm)
	Slot pitch	32 (mm)
	Slot width	18 (mm)
	Number of turns/phase	180
	Iron material	D23

Piston	Permanent magnet's height	4 (mm)
	Permanent magnet's width	36 (mm)
	Permanent magnet's material	NdFe35
	Iron material	D23
	Piston's diameter	275 (mm)

Air gap	Air gap width	0.5 (mm)

## References

[1] Falnes J (2007). A review of wave-energy extraction. *Marine Structures*.

[2] Falcão AFDO (2010). Wave energy utilization: a review of the technologies. *Renewable & Sustainable Energy Reviews*.

[3] Drew B, Plummer AR, Sahinkaya MN (2009). A review of wave energy converter technology. *Proceedings of the Institution of Mechanical Engineers A*.

[4] Zabihian F, Fung AS (2011). Review of marine renewable energies: case study of Iran. *Renewable and Sustainable Energy Reviews*.

[5] Lei H, Hu M, Liu J, Liu C, Zhong W (2013). Design and analysis of a linear hybrid excitation flux-switching generator for direct drive wave energy converters. *Advances in Mechanical Engineering*.

[6] Oğuz Y, Güney İ, Çalık H (2013). Power quality control and design of power converter for variable-speed wind energy conversion system with permanent-magnet synchronous generator. * The Scientific World Journal*.

[7] Chen Z, Yu H, Hu M, Meng G, Wen C (2013). A review of offshore wave energy extraction system. *Advances in Mechanical Engineering*.

[8] Nebel P (1992). Maximizing the efficiency of wave-energy plants using complex-conjugate control. *Proceedings of the Institution of Mechanical Engineers, Part I: Journal of Systems and Control Engineering*.

[9] Salter SH, Taylor JRM, Caldwell NJ (2002). Power conversion mechanisms for wave energy. *Proceedings of the Institution of Mechanical Engineers M: Journal of Engineering for the Maritime Environment*.

[10] de la Villa Jaén A, García-Santana A, El Montoya-Andrade D (2014). Maximizing output power of linear generators for wave energy conversion. *International Transactions on Electrical Energy Systems*.

[11] Falnes J (2002). *Ocean Waves and Oscillating Systems*.

[12] Yeung RW (1981). Added mass and damping of a vertical cylinder in finite-depth waters. *Applied Ocean Research*.

[13] Sabuncu T, Calisal S (1981). Hydrodynamic coefficients for vertical circular cylinders at finite depth. *Ocean Engineering*.

[14] Xu L, Ji J, Liu G, Du Y, Liu H (2014). Design and analysis of linear fault-tolerant permanent-magnet vernier machines. *The Scientific World Journal*.

[15] Ji J, Yan S, Zhao W, Liu G, Zhu X (2013). Minimization of cogging force in a novel linear permanent-magnet motor for artificial hearts. *IEEE Transactions on Magnetics*.

[16] Ahmad ME, Lee HW, Nakaoka M (2006). Detent force reduction of a tubular linear generator using an axial stepped permanent magnet structure. *Journal of Power Electronics*.

[17] Boldea I, Nasar S (1997). *Linear Electric Actuators and Generators*.

[18] Marufuzzaman M, Reaz MBI, Rahman LF, Chang TG (2014). High-speed current *dq* PI controller for vector controlled PMSM drive. *The Scientific World Journal*.

[19] Wu F, Zhang X, Ju P, Sterling MJH (2008). Modeling and control of AWS-based wave energy conversion system integrated into power grid. *IEEE Transactions on Power Systems*.

[20] Beirão P, Valério D, Da Costa JS Linear model identification of the Archimedes Wave Swing.

